# Supplementary material for: Validation of a Food Frequency Questionnaire: VioScreen-Allergy
Source: Nutrients. 2024 Nov 2;16(21):3772. doi: 10.3390/nu16213772 (PMC11547440; doi:10.3390/nu16213772)
Supplement: Supplementary file 1 [file nutrients-16-03772-s001.zip › nutrients-3269637-supplementary.pdf]

Table S1. Comparison of included and excluded cohorts

	Excluded (N=28)	Included (N=25)	Overall (N=53)	p value
**Age** Median (Q1,Q3)	27.7 (24.0, 32.1)	32.3 (28.2, 37.3)	30.7 (25.4, 34.8)	0.008
**What is your current relationship status?**				0.67
Married	13 (46.4%)	15 (60.0%)	28 (52.8%)	
Unmarried	13 (46.4%)	10 (40.0%)	23 (43.4%)	
Other	1 (3.6%)	0 (0.0%)	1 (1.9%)	
Prefer not to answer	1 (3.6%)	0 (0.0%)	1 (1.9%)	
**Pregnant?**	12 (42.9%)	1 (4.0%)	13 (24.5%)	0.001
**What is the highest level of school you have completed?**				0.002
10th grade	1 (3.6%)	0 (0.0%)	1 (1.9%)	
High school graduate - high school diploma or the equivalent (GED)	7 (25.0%)	0 (0.0%)	7 (13.2%)	
Some college, no degree	3 (10.7%)	0 (0.0%)	3 (5.7%)	
Associate degree	3 (10.7%)	1 (4.0%)	4 (7.5%)	
Bachelors degree	7 (25.0%)	17 (68.0%)	24 (45.3%)	
Masters degree	6 (21.4%)	6 (24.0%)	12 (22.6%)	
Professional or Doctorate degree	0 (0.0%)	1 (4.0%)	1 (1.9%)	
Prefer not to disclose	1 (3.6%)	0 (0.0%)	1 (1.9%)	
**What was your total HOUSEHOLD income in the last year?**				0.26
Less than $40,000	5 (17.9%)	1 (4.0%)	6 (11.3%)	
$40,000 to $74,999	8 (28.6%)	6 (24.0%)	14 (26.4%)	
$75,000 or more	9 (32.1%)	14 (56.0%)	23 (43.4%)	
Prefer not to answer	6 (21.4%)	4 (16.0%)	10 (18.9%)	
